# The New Landscape of Cystic Fibrosis in the Era of Highly Effective Cystic Fibrosis Transmembrane Conductance Regulator Modulator Therapy

**DOI:** 10.34763/jmotherandchild.20263001.d-26-00011

**Published:** 2026-09-25

**Authors:** Anna Wołkowicz, Katarzyna Walicka-Serzysko, Justyna Milczewska, Dorota Sands

**Affiliations:** Cystic Fibrosis Department, Institute of Mother and Child, 01-211 Warsaw, Poland; Cystic Fibrosis Centre, Paediatric Hospital, Dziekanow Lesny, 05-092 Łomianki, Poland

**Keywords:** cystic fibrosis, CFTR modulators, elexacaftor, ivacaftor, vanzacaftor

## Abstract

**Background:**

For many years, the management of cystic fibrosis has had a personalised character. We have now entered an era of therapy based on a patient’s specific genotype. Clinical trials have confirmed the efficacy and safety of cystic fibrosis transmembrane conductance regulator modulator therapies, including ivacaftor as monotherapy and triple-drug combinations, such as elexacaftor/tezacaftor/ivacaftor and vanzacaftor/tezacaftor/deutivacaftor. These therapies result in substantial and sustained improvements in lung function, a reduction in the frequency of bronchopulmonary exacerbations, enhanced nutritional status, and an improved quality of life. These clinical outcomes have been correlated with a marked reduction of sweat chloride values.

**Material and methods:**

Early initiation of therapy is crucial to halt disease progression or even reverse disease-related complications, which have even been observed in children aged six to twelve years. Initiating treatment in even younger children before the onset of clinical symptoms offers the possibility of a much later disease manifestation or even absence of symptoms. Nevertheless, careful evaluation of these therapies’ long-term effects on the respiratory system and other organs remains necessary.

**Results:**

According to current knowledge, patients continue to require regular follow-up by a multidisciplinary care team and ongoing adjustment of both modulator and symptomatic therapies to meet individual needs.

**Conclusion:**

As patient life expectancy continues to increase, comorbidities such as arterial hypertension, hypercholesterolaemia, cardiovascular disease, and malignancies will assume greater clinical importance. Selecting optimal therapeutic strategies remains a significant clinical challenge due to the numerous potential interactions between modulators and other medications.

## 1. Introduction

For decades, cystic fibrosis was considered a chronic, progressive, life-shortening disease. The first standards of care were developed in the 1960s. These subsequently evolved in parallel with improved understanding of disease mechanisms and the availability of new medications. Advances in medicine, better understanding of the underlying pathophysiology of the disease, and the introduction of novel therapies resulted in a gradual extension of patient life expectancy. A breakthrough in recent years has been the introduction of therapies targeting the underlying cause of the disease: the defect of the cystic fibrosis transmembrane conductance regulator (CFTR) protein. The CFTR protein is an ion channel for chloride anions, located at the apical surface of secretory epithelium. The development of CFTR modulators in 2012, followed by their progressive optimisation and expansion to increasingly broad patient populations, has fundamentally changed the clinical picture of cystic fibrosis and has created new challenges for both patients and multidisciplinary care teams.

The authors conducted a literature review using the MEDLINE/PubMed database, encompassing English-language publications from 2011 (when the first publication on the in vivo efficacy of a CFTR modulator was reported) to November 2025. The review aimed to identify changes in cystic fibrosis in the era of highly effective CFTR modulator therapy (HEMT), as well as to define new therapeutic challenges and future perspectives for patients.

Cystic fibrosis is the most common autosomal recessive disorder. Pathogenic *CFTR* gene variants can result in abnormal production, transport, or function of the CFTR protein. The protein is involved in the regulation of other chloride and sodium channels, adenosine triphosphate production, and protein phosphorylation [1]. Dysfunction of the CFTR protein leads to the accumulation of thick mucus. In the respiratory tract, this viscous mucus impairs mucociliary clearance, resulting in colonisation with pathogenic flora and chronic inflammation, leading to progressive lung tissue damage and chronic rhinosinusitis. Within the gastrointestinal system, dysfunction of the pancreas, liver, bile ducts, and intestines may occur, potentially resulting in malabsorption syndrome, diabetes, liver failure, hypersplenism, and portal hypertension [2]. Lung disease is usually the most severe manifestation of cystic fibrosis, and respiratory failure remains the leading cause of death [3].

## 2. Genetic basis of cystic fibrosis

The *CFTR* gene was discovered in 1989. To date, more than 2,100 *CFTR* gene variants have been described, resulting in a wide spectrum of phenotypes. The CFTR2 database includes 1,167 variants, of which 1,085 are classified as cystic fibrosis-causing, 55 have uncertain clinical consequences, and 27 are considered non-disease-causing. The impact of cystic fibrosis-causing variants on CFTR protein production, stability, and channel activity is heterogeneous. Seven classes of variants have been distinguished. Variants belonging to classes I–III result in complete loss of chloride channel function (minimal function) and are usually associated with a more severe disease course. In contrast, class IV–VII mutations lead to partially preserved ion channel function (residual function) and most often result in a milder phenotype, characterised by the later onset of symptoms, preserved exocrine pancreatic function, lower sweat test values, slower progression of bronchopulmonary disease, and reduced susceptibility to respiratory tract infections with pathogenic flora [4].

This classification of variants has gained particular importance in the era of disease-modifying therapies. It forms the basis for the stratification of treatments targeting specific CFTR protein defects. Currently, however, the greatest clinical relevance lies in the identification of variants that do not result in protein production (class I), as CFTR modulators are not effective in these cases [5].

## 3. CFTR modulators

As a result of successive management changes over the past six decades, cystic fibrosis is no longer a cause of death in early childhood. However, it is still considered a chronic disease [5]. Until recently, therapeutic strategies in cystic fibrosis were based exclusively on symptomatic treatment tailored to the individual patient’s needs (best supportive care).

CFTR modulators are the first approved medications that directly improve CFTR ion channel function. Among the agents that have successfully completed the regulatory approval process, two main classes are distinguished: potentiators and correctors. HEMT is of particular importance for use in patients carrying at least one variant that results in CFTR protein production (a mutation other than class I). Current HEMTs include ivacaftor as monotherapy, elexacaftor/tezacaftor/ivacaftor (ELX/TEZ/IVA), and vanzacaftor/tezacaftor/deutivacaftor (VAN/TEZ/DIV).

### 3.1 Potentiators

Potentiators improve the ion channel function of the CFTR protein. The potentiator is only effective if the CFTR protein is present in the cell membrane; therefore, it is intended for patients carrying at least one class III or class IV mutation. Ivacaftor is the first, and to date, the only potentiator approved for use as monotherapy.

This drug was initially approved for patients with the class III G551D variant. Pre-registration clinical trials demonstrated a remarkable improvement in sweat test (a mean reduction in sweat chloride concentration of 48.1 mmol/L from baseline), an increase in ppFEV_1_ (percentage points forced expiratory volume) of 10 percentage points, a reduction of 55-percentage points in the rate of pulmonary exacerbations, as well as an improvement in nutritional status. In all study participants, sweat chloride concentrations decreased below the diagnostic threshold for cystic fibrosis (<60 mmol/L) [6]. Subsequent studies confirmed the efficacy of ivacaftor in younger patients with other gating mutations [7] and in those with residual function mutations [8,9]. Improvement in pancreatic function was also observed in infants between four and twelve months of age who had been initially diagnosed with exocrine pancreatic insufficiency [10].

In a six-year, multicentre, prospective observational study in patients carrying the G551D variant, sustained clinical benefits were confirmed, including the maintenance of improved FEV_1_ (forced expiratory volume) values, a reduced number of pulmonary exacerbations, better quality-of-life scores, and a lower prevalence of *Pseudomonas aeruginosa* infections. The age-related decline in ppFEV_1_ in these patients was comparable to that observed in the healthy population, amounting to approximately one to two percentage points per year, whereas in children with cystic fibrosis receiving symptomatic treatment alone, the decline averages two to three percentage points per year. Persistently lower sweat chloride values were also observed [11]. Long-term effectiveness has additionally been confirmed in real-world studies based on patient registry data [12].

Ivacaftor is a highly effective drug with a favourable safety profile, as confirmed by long-term post-authorisation studies. However, it can be used in a relatively small group of patients, since the gating and residual function mutations are relatively rare [3].

Deutivacaftor is a once-daily successor to ivacaftor with comparable efficacy, but it has not been approved for use as monotherapy [13].

### 3.2 Correctors

Correctors, as molecular chaperones, assist the folding of the CFTR protein, stabilising its conformation and facilitating its transport and insertion into the cell membrane. The first approved corrector was lumacaftor in combination with ivacaftor. This therapy is intended for patients who are homozygous for the F508del mutation. According to the CFTR2 mutation database and the most recently published Annual Report of the European Cystic Fibrosis Society Patient Registry (ECFSPR) [3], F508del is the most common variant in individuals with cystic fibrosis. It is estimated that approximately 70–80% of patients have the F508del mutation on at least one allele, with around 40% being homozygous for this variant. This mutation, like several others, exhibits features of different classes. It is classified as a class II mutation, but under certain conditions, it also reveals the characteristics of classes III and VI.

Efficacy of lumacaftor in combination with ivacaftor (LUM/IVA) has been demonstrated in randomised, double-blind, placebo-controlled clinical trials. Clinical benefits included a 30–38-percentage points reduction in the overall rate of pulmonary exacerbations, a 56-percentage points reduction in exacerbations requiring intravenous antibiotic therapy, improvement in nutritional status (weight gain of 1,23–1,57 kg), enhanced quality of life, and an increase in ppFEV_1_ of approximately 2,6–4 percentage points. Unfortunately, initiation of therapy was associated with a significantly higher incidence of respiratory adverse events, such as dyspnoea and chest tightness [14].

According to multicentre studies conducted in patients with advanced bronchopulmonary disease (FEV_1_ <40%), more than 50% of participants reported respiratory adverse events. These were usually transient; however, in approximately 30% of patients, they led to discontinuation of therapy. Importantly, the study confirmed the efficacy of LUM/IVA in this population, with a mean increase in ppFEV_1_ of two percentage points after one month of treatment and three percentage points after three months of treatment. In 30% of patients, the increase reached five percentage points, and in 13% of patients, ppFEV_1_ improved by as much as ten percentage points. Improvement in nutritional status was also observed [15].

The greatest improvement in lung function was observed in patients with FEV_1_ between 40% and 90%. Patients with FEV_1_ above 40% experienced the greatest reduction in the frequency of intravenous antibiotic therapy. Nutritional status improvement was observed in all groups, regardless of lung capacity [16].

Tezacaftor is a second-generation corrector based on the chemical structure of lumacaftor. In clinical trials, tezacaftor in combination with ivacaftor (TEZA/IVA) demonstrated comparable efficacy to lumacaftor with a lower incidence of adverse events and fewer drug-to-drug interactions. Treatment was associated with an improvement in ppFEV_1_ of approximately 3,4 percentage points, a lower rate of bronchopulmonary exacerbations, and an improvement in quality-of-life scores compared with the placebo group. Mean sweat chloride concentration decreased by approximately 10 mmol/L. The frequency of adverse events was similar in the treatment and control groups, and no increased incidence of respiratory symptoms was observed [17].

In patients carrying the F508del mutation in combination with a class IV or V variant (i.e., mutations associated with preserved residual function), treatment with TEZA/IVA was more effective than in F508del homozygotes. This combination was also more effective than ivacaftor monotherapy [18]. Moreover, the therapy was well tolerated even in patients who had previously discontinued LUM/IVA due to adverse events [19].

The addition of a next-generation corrector, elexacaftor, to the previously established combination of tezacaftor and ivacaftor (ELX/TEZ/IVA) brought unexpectedly favourable results in both F508del homozygotes (an increase in ppFEV_1_ of approximately ten percentage points) and heterozygotes for this mutation (an increase in ppFEV_1_ of approximately 14 percentage points) [20]. These findings were subsequently confirmed in further clinical trials.

At 24 weeks after treatment initiation in patients carrying the F508del *CFTR* gene mutation on one allele and a variant with preserved residual function on the other allele, a sustained increase in ppFEV_1_ of approximately 14 percentage points was observed, along with a 63-percentage points reduction in the number of pulmonary exacerbations and a decrease in sweat chloride concentration of nearly 42 mmol/L from baseline. The quality-of-life questionnaire revealed a remarkable improvement of 14 percentage points from baseline, an increase not previously reported with other therapies. The triple combination was well tolerated, with no new adverse events reported, when compared with the TEZ/IVA therapy [21]. Comparable treatment effects were also observed in patients homozygous for the F508del mutation [22]. A similar result was obtained in studies conducted in Poland [23].

A recent Phase Three, double-blind, randomised, placebo-controlled study confirmed the efficacy and safety of ELX/TEZ/IVA in adult and paediatric patients with cystic fibrosis carrying a non-F508del *CFTR* gene mutation responsive to ELX/TEZ/IVA.

In December 2024, the Food and Drug Administration also approved the registration of another triple-combination therapy, consisting of vanzacaftor, tezacaftor, and deutivacaftor (VAN/TEZ/DIV). This formulation includes a next-generation potentiator (vanzacaftor) and a once-daily corrector (deutivacaftor). In April 2025, the therapy was also approved by the European Medicines Agency for use in patients aged six years and older with at least one gene variant other than a class I *CFTR*. This regimen demonstrates efficacy comparable to that of ELX/TEZ/IVA, with a similar safety profile and the advantage of a once-daily dosing schedule.

Pre-registration studies showed that switching patients previously treated with ELX/TEZ/IVA to the novel triple combination VAN/TEZ/DIV did not result in deterioration of lung function (FEV_1_) and was associated with a slight reduction (8 mmol/L)in sweat chloride concentration [24,25]. Based on in vitro studies, it has also been suggested that the VAN/TEZ/DIV combination may be effective in 31 *CFTR* gene variants that do not respond to ELX/TEZ/IVA [24].

## 4. Impact of personalised therapy on the clinical profile of cystic fibrosis

According to data from the ECFSPR and the Cystic Fibrosis Foundation Patient Registry, since the approval and widespread implementation of CFTR modulators — particularly HEMT — numerous positive outcomes have been achieved. The most prominent benefits are pulmonary. A significant increase in mean FEV_1_ values has been reported across all age groups older than six years. Pulmonary function, expressed as FEV_1_% predicted, has shown a consistent upward trend over time, with a clear improvement following the introduction of CFTR modulators [26]. In parallel, a marked reduction in the number of patients requiring lung transplantation has been observed. In the United States, the number of lung transplants decreased from 249 in 2019 to 61 in 2024 [27,28].

Based on radiological lung assessments, such as magnetic resonance imaging and high-resolution computed tomography, initiation of HEMT has been shown to improve mucus plugging and bronchial wall thickening and, in some cases, leads to partial or even complete resolution of bronchiectasis [29,30].

The introduction of HEMT has also been associated with increased bacterial diversity in patients’ sputum, accompanied by a reduction in pathogenic flora, such as *Pseudomonas aeruginosa*, *Stenotrophomonas maltophilia*, methicillin-resistant *Staphylococcus aureus*, *Burkholderia cepacia* complex, and nontuberculous mycobacteria. However, in most patients, eradication of these pathogens is not complete. In some individuals, they remain detectable in sputum at lower titres, while in others they are detectable only by Polymerase Chain Reaction-based methods [27,31]. Clinical monitoring becomes more challenging due to reduced airway secretions and the markedly increased difficulty in obtaining sputum samples for microbiological analysis [32].

In many patients, improvement in nutritional status has been observed; however, a significant increase in the prevalence of overweight and obesity has also been reported, particularly in the adult population [27,32,33].

Individual reports suggest a potential effect of ELX/TEZ/IVA and ivacaftor used as monotherapy on exocrine pancreatic insufficiency, when treatment is initiated in younger children [32,34]. This effect has not been observed in older children or in adults [32]. In some patients with cystic fibrosis-related diabetes, improvement in glycaemic control and the possibility of reducing insulin doses have been reported. Data from patient registries show a lower incidence of newly diagnosed diabetes [27].

A multidirectional improvement in health status resulted in a significant increase in predicted survival [35]. The estimated average life expectancy of patients who started ELX/TEZ/IVA treatment between the ages of 12 and 17 is 82.5 years, which is 45.4 years longer compared to best supportive care treatments [35,36]. This constitutes an undeniable therapeutic success. Despite this, increased life expectancy is also associated with an increasing number of adults with cystic fibrosis and the need to reorganise and expand long-term care for these patients.

With increasing age, the prevalence of extrapulmonary complications of cystic fibrosis rises, including liver disease, cystic fibrosis-related diabetes, osteoporosis, cardiovascular disease, arterial hypertension, and malignancies [28]. Consequently, comprehensive care requires access to specialists from multiple disciplines, as well as an enhanced role for primary care. In the context of multimorbidity, there is also an increased risk of drug-to-drug interactions involving CFTR modulators and the need for dose adjustments [36].

In some patients, transient and self-limiting adverse events have been observed, such as increased airway secretions, rash, abdominal pain, and headache. In others, features of hepatocellular damage have been reported. Therefore, treatment requires regular monitoring and, if necessary, dose adjustment.

Some patients experience neuropsychiatric adverse effects, including low mood, anxiety, sleep disturbances, cognitive impairment, and suicidal thoughts or attempts. It is therefore necessary to assess mental health status before treatment initiation and to carefully monitor the status during therapy [30].

The longer life perspective is associated with the need for planning for the future — this includes an appropriate education and future job preparation. Worldwide, employment rates for people with cystic fibrosis currently range between 44% and 86%. In the future, the proportion of economically independent patients is likely to increase. Access to career counselling will therefore become crucial [37].

One of the major challenges of these drugs is the increasing frequency of pregnancies, including unplanned ones, in women treated with CFTR modulators. There is limited data on the safety of these drugs during pregnancy. While it is known that these drugs cross the placenta, their effects on the developing foetus remain unclear. To date, cases of lens opacities have been reported. Further studies are required to assess the long-term impact of in utero exposure to CFTR modulators on foetal and child development. On the other hand, discontinuation of therapy during pregnancy may be associated with deterioration in maternal health, thereby increasing risks for both the mother and the child. Consequently, preconception counselling and careful family planning are of critical importance [37].

Following in utero exposure to variant-specific therapy, a case report described an infant with cystic fibrosis who had normal immunoreactive trypsinogen levels at birth. When reviewing newborn screening results, clinical teams should be alert to the possibility of false-negative screening outcomes in this context [37].

Another issue under ongoing discussion is the optimal timing for initiation of therapy. Current evidence suggests that starting treatment at a younger age slows disease progression by reducing pulmonary exacerbations and preserving lung function, which was not fully restored by initiating therapy at older ages. This effect has been clearly proven in patients treated with ivacaftor as monotherapy [38]. On the other hand, the long-term consequences of HEMT remain unknown.

It should be emphasised that the remarkable benefits of HEMT have been observed in patients who continued their standard treatment. According to the “Standards of Care for People with Cystic Fibrosis Receiving CFTR Modulator Therapy” issued by the ECFSPR, as well as their adaptation to Polish clinical practice by the Polish Cystic Fibrosis Society, interdisciplinary management remains the cornerstone of care. This approach focuses on achieving optimal nutritional status, appropriate pancreatic enzyme supplementation, regular physiotherapy, prevention and early treatment of pulmonary exacerbations, and active surveillance for and treatment of infections caused by prognostically adverse pathogens, such as *Pseudomonas aeruginosa* [33,34,39].

In 2023, approximately 30% of eligible patients in Europe were not receiving any CFTR modulator therapy [3]. Moreover, in 15–20% of patients, CFTR modulator treatment is currently not feasible, particularly in those with class I variants in which mRNA is not produced [40]. These patients will continue to require special care from a multidisciplinary team while awaiting additional therapeutic options, including gene therapy, which could constitute the next stage of treatment in the future.

### Key points

1. Highly effective CFTR modulator therapies (HEMT) are: ivacaftor as monotherapy and triple-drug combinations, such as elexacaftor/tezacaftor/ivacaftor and vanzacaftor/tezacaftor/deutivacaftor).
2. HEMT results in substantial and sustained improvements in lung function, a reduction in the frequency of bronchopulmonary exacerbations, enhanced nutritional status, and an improved quality of life.
3. Early initiation of therapy is crucial to halt disease progression or even reverse disease-related complications.
4. Patients with cystic fibrosis should continue to be cared for by a multidisciplinary team at a centre specialising in the treatment of cystic fibrosis.
